# The relationship between exercise addiction, eating disorders, and insecure attachment styles among recreational exercisers

**DOI:** 10.1186/s40337-023-00855-3

**Published:** 2023-08-07

**Authors:** Dalit Lev Arey, Adi Sagi, Asaf Blatt

**Affiliations:** 1 School of Psychology, The Academic College of Tel Aviv-Yaffo, Tel Aviv, Israel; 2 School of Behavioral Sciences, College of Management Academic Studies, Rishon LeZion, Israel

**Keywords:** Exercise addiction, Exercise dependence, Eating disorders, Addiction, Pathological exercise, Recreational exercisers, Insecure attachment

## Abstract

**Background:**

Exercise addiction (EA) and eating disorders (ED) frequently co-occur in both professional and amateur athletes, with up to 48% of individuals with EA also exhibiting symptoms of ED. Furthermore, pathological attachment styles have been linked to both EA and ED. The current study aimed to explore the unique association between types of insecure attachment styles (i.e., anxiety or avoidance) and EA and ED.

**Method:**

Four hundred and five Israelis (199 women, 206 men) who were recreational exercisers (i.e., exercised at least four hours a week for at least one year) with ages ranging from 18 to 78 (*M* = *38, SD* = *12.31*) completed a set of questionnaires, including the Eating Attitudes Test (EAT-26), Exercise Addiction Inventory, and the Experiences in Close Relationship Scale. Path analysis was used to simultaneously examine the associations of attachment anxiety and avoidance with EA and ED symptoms. Attachment anxiety and avoidance were specified as independent variables predicting ED and EA symptoms and were entered into the analysis as two parallel dependent variables.

**Results:**

The results of the study indicate that attachment anxiety is positively associated with symptoms of EDs, while the association between attachment anxiety and symptoms of EA is not significant. On the other hand, attachment avoidance shows a positive association with symptoms of exercise addiction, but no significant association with symptoms of EDs is found.

**Conclusions:**

These results imply that the anxious attachment regulation strategy is highly associated with body image concerns. Furthermore, individuals characterized by avoidance attachment manifest regulation strategies through excessive exercise. Scholars and practitioners could use these results to examine dispositional risk factors for insecure attachment styles and to assess specific pathologies among the population of recreational exercisers. The study also discusses limitations, future directions, and implications in detail.

## Abstract (highlights)

The present study aimed to investigate the relationship between exercise addiction, eating disorders, and pathological attachment styles among habitual exercisers. Approximately half of individuals addicted to exercise also exhibit maladaptive eating behaviors. Insecure attachment styles have been associated with these two pathologies. However, the exact nature of this relationship is not fully understood and requires further research. A total of 405 habitual athletes who exercised for a minimum of four hours per week for at least one year were recruited from social media groups dedicated to exercise, as well as through ads posted at fitness centers and with professional trainers. Participants completed three psychological inventories, including measures of eating disorder symptoms, exercise addiction, and attachment styles. Results indicated that attachment anxiety was solely related to symptoms of maladaptive eating behaviors, while attachment avoidance was only associated with symptoms of exercise addiction. Findings suggest that individuals with anxious attachment may be more prone to body image concerns, whereas those with avoidance attachment may be more likely to engage in compulsive exercise behaviors. These results have implications for scholars and practitioners in terms of examining dispositional risk factors for insecure attachment styles and assessing specific pathological and addictive behaviors among recreational exercisers.

## Introduction

### Exercise addiction

With the growing popularity of exercise and its impact on individuals’ lives, physical activity and exercise are widely recognized as having significant benefits for physical health, including improved overall health and disease prevention [52, 79]. Engaging in regular physical activity has also been associated with numerous positive effects on mental health, including reduced symptoms of depression and anxiety, boosted mood and emotional well-being, and increased resilience to stress [26, 65]. Recent research, however, has highlighted the potential negative physiological and psychological symptoms associated with excessive exercise, which may contribute to the development of exercise addiction (EA) [72].

EA is characterized by uncontrolled, urge-driven exercise that becomes dysfunctional and harmful to the individual [45]. The behavior is often characterized by a progressive increase in exercise volume and frequency, making it challenging to control exercise behavior despite negative physical and mental consequences [11, 25]. While EA is not a widespread phenomenon, occurring in only 0.3–3% of the general population and 3–14% of the exercising population [11, 75], it could have severe physical and mental consequences for those affected, including injuries and social isolation [13, 73].

EA has been described using different terms such as exercise dependence, compulsive exercise, and obligatory exercise. For the purposes of this study, we use the term EA as it encompasses both features of dependence and compulsion [72]. Hausenblas and Symons Downs [39] developed a set of criteria for EA based on modifications of the Diagnostic and Statistical Manual of Mental Disorders, 4th edition (DSM-IV; American Psychiatric Association [5]), criteria for substance dependence. These criteria include six symptoms of EA, with the presence of some or all these symptoms indicating a likelihood of having become addicted. The six symptoms are: (1) salience, where exercise becomes the most important thing in people's lives, consuming most of their time and dominating their thinking; (2) mood modification, where exercise is used to facilitate a distinct and consistent change in mood state; (3) tolerance, where individuals require longer and more intense periods of exercise to achieve previously acquired effects; (4) withdrawal symptoms, where unpleasant physiological and psychological effects occur when people cannot maintain their regular level of activity or stop altogether; (5) conflict, where people experience inter- and intrapersonal conflicts because of a high level of engagement in the behavior; and (6) relapse, where addictive patterns of behavior tend to reemerge [39].

To assess EA risk, an individual’s exercise engagement must be measured, including exercise frequency and history [54]. These factors are critical in selecting participants for research examining EA. Previous studies have suggested that exercising at least four times a week (i.e., recreational exercisers) and having an exercise history longer than one year represent the minimum standards for the investigation of EA [63]. These criteria ensure that participants have a high level of exercise engagement and are likely to be at greater risk of developing EA, thus enabling researchers to examine the phenomenon more closely.

### The relationship between EA and ED

The relationship between EA and EDs has also been the focus of research. EA symptomatology often co-occurs with symptoms of other disorders, where EDs are most commonly associated with EA [11]. EDs are mental health conditions characterized by abnormal or disturbed eating behaviors, thoughts, and emotions, which could result in negative physical and psychological consequences [6]. Herein, among individuals with EA, it has been reported that between 39 and 48% are also at risk for developing EDs [22]. Indeed, many of the reported characteristics of EDs are also prevalent among people with EA [76], such that excessive exercise reportedly affects 40–50% of patients with anorexia nervosa and 20–24% of patients with bulimia nervosa [67]. In a previous study, 28% of ED patients were described as “compulsive exercisers” [16]. Various studies have shown that EA is three-and-a-half times more prevalent among people with an ED than among those without an ED [75].

The co-occurrence of EA and EDs is frequently observed among athletes participating in various types of professional and amateur sports. In a recent study by Godoy-Izquierdo and colleagues [37], the need for further quantitative research investigating the characteristics and correlates of this comorbidity was highlighted. One mechanism that may underly the occurrence of both psychological conditions is an insecure attachment (IA) style, which was previously associated with several other psychological pathologies (e.g., sex addiction, depression, anxiety disorders). The IA style has also been linked to EDs, but results were conflicting [23]. For example, several studies have shown a relationship between attachment styles and EDs [19, 82], while others have shown mixed findings [62] or non-significant relationships [71]. Moreover, only one study has tested the association between attachment and EA, with no significant results [53].

### Insecure attachment, addictions, and disorders

Attachment theory suggests that individuals acquire coping mechanisms for negative emotions and distress through their experiences with attachment figures [59]. Central to this theory is the concept of attachment styles, which encompass an individual's cognitive and emotional perceptions of themselves and others in intimate relationships. These styles are shaped by repeated interactions with attachment figures and involve security levels, coping strategies for negative experiences, and frameworks for regulating affect and expressing attachment needs [2, 40].

As mentioned earlier, IA styles have been identified as correlating with a range of addictions and disorders, including exercise addiction (EA) and eating disorders (EDs) [21, 47]. A comprehensive meta-analysis of 34 studies revealed that IA precedes the development of substance abuse problems, regardless of the specific psychoactive substance involved [29]. Furthermore, IA has been associated with behavioral dependencies such as video game addiction and gambling disorder [27], as well as anxiety and borderline personality disorders [15].

These connections are believed to be mediated by deficits in emotion regulation, as individuals with IA often employ non-constructive methods to regulate their emotions and underutilize social support [29, 58]. Moreover, IA has been found to have a positive correlation with stress, increasing vulnerability to the development of pathological and addictive disorders [3]. Individuals with IA may exhibit dysfunctional emotion regulation, leading to a greater inclination to rely on maladaptive resources to fulfill their attachment needs [7, 15].

These findings align with predisposition models that propose vulnerability factors play a causal role in the development of various psychological pathologies [77]. Additionally, self-regulation (SR) theory suggests that deficiencies in emotional self-regulation mechanisms contribute to the increased prevalence of psychological disorders [4].

### Individual patterns of attachment, addictions, and disorders

**Fig. 1:**
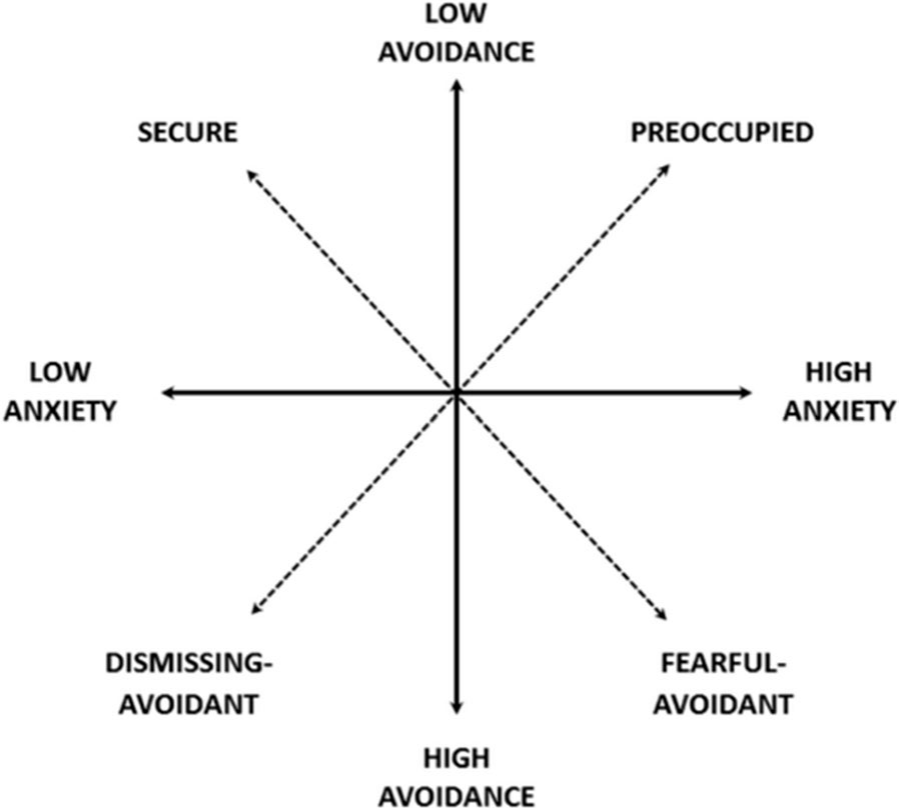
The two-dimensional model (four-categories) of adult attachment. The two-dimensional model (four-categories) of adult attachment. Note: The combination of two dimensions (high/low avoidance; high/low anxiety) together constitute four separate attachment styles. Each one represents different attachment patterns

Building on the idea of vulnerability factors and SR deficiencies, different types of attachment styles have been identified that may contribute to these issues. Ainsworth and colleagues [2] originally defined three attachment styles: secure attachment and two insecure attachment styles—anxious and avoidant. Hazan and Shaver [40] extended Ainsworth’s research to adult relationships and conceptualized attachment style using two distinctive dimensions, namely, attachment anxiety and attachment avoidance (Fig. 1). The anxiety dimension reflects the extent to which an individual seeks intimacy with others, whereas the avoidance dimension encompasses a fear of intimacy and difficulty in trusting others. Individuals with low levels of one or both dimensions are assumed to have an IA style.

People implement different SR strategies based on their attachment style [19]. For example, individuals with avoidant attachment have negative attitudes towards others, mistrust others, and have a propensity for isolation and loneliness. Consequently, they may suppress or deactivate emotional reactions to interpersonal threats [66]. In other words, they use maladaptive SR strategies that suppress emotions, repudiate stress, or divert attention away from emotion-eliciting stimuli [18]. Conversely, individuals with anxious attachment style who crave close relationships may engage in strategies that sustain or even aggravate their distress [66]. Since these individuals are extremely afraid of rejection, they tend to hold their anger in and re-direct it towards themselves. This unhealthy SR strategy can trigger feelings of resent towards a partner, as well as self-criticism, sadness, and depression [69]. Differences in SR strategies may also be related to the use of various substances and maladaptive behaviors to alleviate high levels of stress [10].

Research has suggested that there is a positive relationship between EDs and anxious attachment. A meta-analysis of 70 studies (*N* = 19,470) conducted by Faber and colleagues [28] found a positive moderate association between higher levels of anxiety attachment and the manifestation of unhealthy eating behaviors. However, results regarding the link between EDs and avoidance attachment have been inconsistent. For instance, some studies have found a positive association between avoidance attachment and EDs [46], while others have not found a significant relationship [78].

In the case of EA, a systematic review examining the relationship between exercise and loneliness, a pivotal component of avoidance attachment, yielded ambiguous results [64]. While exercise has been found to decrease loneliness, physical inactivity may over time lead to feelings of loneliness. Lukács and colleagues [54] also found that lonely individuals are more likely to report exercise withdrawal and uncontrolled exercise behaviors. Furthermore, in a study examining exercise habits based on adult attachment, individuals with an avoidant attachment style reported exercising significantly more frequently and for longer durations than those with an anxious attachment style [20].

### Purpose and hypotheses

There is some evidence suggesting a positive link between anxious attachment and an increased risk for developing EDs. However, further research is needed to confirm this relationship and to explore the potential association between attachment style and EA. Given the serious consequences and growing prevalence of these pathologies, it is critical to understand the underlying mechanisms and risk factors. Ongoing investigation into the relationship between attachment style and pathological and addictive disorders is essential for advancing our understanding and improving prevention and treatment efforts. Based on the available evidence, we hypothesized the following:

H1: Anxious attachment will be linked to ED but not to EA.

H2: Avoidance attachment will be linked to EA but not to ED.

## Methods

### Participants

The study involved a sample of 405 Israeli recreational exercisers, including 199 women and 206 men, with an age range of 18–78 years *(M* = 38.00*, SD* = 12.31). To increase the likelihood of sampling individuals with EA, we implemented stricter criteria for exercise duration and frequency. We used the criterion suggested by Huang and colleagues [43] of exercising for at least one year as a measure of exercise duration. Research has shown that exercise is a habit that requires significant effort to establish and can take several months to become a routine behavior [32, 50]. Additionally, only around 50% of individuals who initiate an exercise program continue with it for six months, and even fewer continue for a full year [1].

Regarding exercise frequency, McKinney et al. [55] categorized habitual exercisers into two groups based on their exercise frequency. The first group comprised individuals who engage in at least 2.5 h of exercise per week for health and fitness, while the second group comprised recreational exercisers who engage in at least 4 h of exercise per week. The World Health Organization (WHO) recommends a minimum of 150 min of moderate-intensity aerobic activity per week, along with muscle-strengthening activities on at least two days per week. Our study aimed to identify individuals who engage in even more physical activity than the recommended minimum by selecting recreational exercisers who engage in a minimum of 4 h of exercise per week.

Participants were recruited between May and September 2022 by the first and second authors of this paper through social media groups (e.g., Facebook) focused on exercise, advertisements posted at fitness centers and professional trainers.

### Measures

*Demographics* Demographic data were collected (age, gender, marital status, income level, and education level). In addition, participants were asked to select their main form of exercise from a list of options (running, swimming, biking, triathlon, gym workouts, other), the number of hours they exercised per week, and the length of time they had been exercising regularly (1–2 years, 2–5 years, 5–10 years, more than 10 years). The most common forms of exercise were as follows: running *(n* = 145*)*, triathlon (*n* = 54), gym workouts (*n* = 48), swimming (*n* = 25), aerobics (*n* = 23), and biking (*n* = 15). Moreover, 93 participants chose the “other” option and mentioned various forms of exercise (e.g., boxing, yoga, basketball, CrossFit) as well as combinations of the above options. Participants reported devoting an average of 7.40 h (*SD* = 4.22) per week to exercise.

*Exercise addiction inventory* Exercise addiction was assessed using the Hebrew version of the Exercise Addiction Inventory (EAI; Weihrauch [81]). The EAI, which was developed in 2004 [74] from a theoretical model of behavioral addictions [17], is a six-item self-report survey. Participants respond by means of a five-point Likert-type scale. A total score of 24–30 indicates a high risk of addiction. In the current sample, the internal reliability of the scale was adequate (*Cronbach’s* α = 0.73).

*Eating attitudes* Eating attitudes were measured using the Hebrew version of the Eating Attitudes Test (EAT-26; [9], which was designed to screen for ED symptoms [33]. The EAT-26 contains 26 items that participants answer on a 4-point Likert-type scale. The test includes three subscales to evaluate the impact of environmental and social factors on food ingestion: 1 diet, related to pathological rejection of foods with high-calorie content and concerns about physical appearance, 2 bulimia and concerns about foods, referring to episodes of compulsive eating followed by purgative behavior for body weight loss/control; 3 oral self-control, which designates self-control concerning food. In this study, we computed the sum score of all items that had a high level of internal reliability (α = 0.88). Scores of 20 or higher on this measure indicate a tendency towards maladaptive eating behaviors.

*Attachment* Adult attachment was evaluated using the Experiences in Close Relationship Scale (ECR; [14]. We used the Israeli version that was translated and validated by Mikulincer and Florian [56] into Hebrew. The ECR is a 36-item self-report measure scored on a 7-point Likert type scale. It includes two subscales designed to assess the avoidance (18 items) and anxiety (18 items) dimensions of adult attachment. Higher scores on the avoidance and anxiety subscales indicate higher levels of attachment anxiety and attachment avoidance, respectively. In the present sample, Cronbach’s α was 0.87 for the avoidance subscale and 0.90 for the anxiety subscale.

### Procedure

The participants completed the questionnaires mentioned above using the Qualtrics platform [70], which can be accessed via any computer or mobile phone connected to the internet. Once participants' inclusion criteria were verified, the collected data were analyzed using SPSS (Statistical Package for Social Sciences, V.21) and Mplus software.

### Statistical analysis

The associations between attachment anxiety and avoidance and ED symptoms and EA symptoms were analyzed using a path analysis in Mplus version 8.3 [61]. Attachment anxiety and avoidance were treated as independent variables predicting ED symptoms and EA symptoms (measured using EAT-26 and EAI sum scores, respectively), which were entered into the analysis as two parallel dependent variables.

During the analysis, the error distributions of the dependent variables were examined to check for any violations of assumptions. It was found that there was a slight positive skew in the EAT-26 scores. To address this issue, a log10 transformation was applied to the EAT-26 score, and the analysis was repeated. The results of the analysis with the transformed EAT-26 scores were consistent with the original analysis. Therefore, the results reported in the study are based on the original EAT-26 scores.

## Results

Descriptive statistics and bivariate correlations between the main study variables are provided in Table 1. The variables were positively intercorrelated, with greater attachment anxiety and avoidance related to greater EA and ED symptoms.

**Table 1:** Descriptive statistics and bivariate correlations between study variables

	1	2	3	4
1. Attachment avoidance				
2. Attachment anxiety	.22***			
3. Eating disorder symptoms	.13**	.37***		
4. Exercise addiction symptoms	.24***	.14**	.37***	
Mean	3.06	3.21	12.05	28.43
SD	0.90	1.12	10.65	6.50

**p < .01, ***p < .001

A path analysis was specified to examine the unique associations of attachment anxiety and avoidance with ED and EA symptoms.

**Fig. 2:**
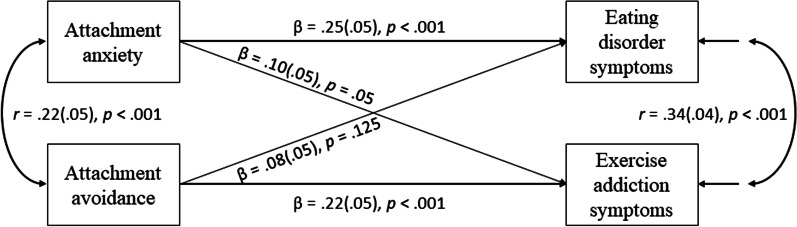
Path Analysis model for maximum likelihood estimates for attachment anxiety and avoidance predicting ED and EA. Note: The model is saturated (DF = 0)

Standardized maximum likelihood estimates for this model are presented (see Fig. 2). As predicted, attachment anxiety was positively associated with ED symptoms, but its association with EA symptoms failed to reach significance. In contrast, attachment avoidance was positively associated with EA symptoms, but not with ED symptoms.

To examine whether the associations between attachment orientations and each type of symptoms are confounded by the other type of symptoms, we conducted two additional regressions, each one regressing one symptom type on attachment anxiety and avoidance, while controlling for the other symptom type. As seen in Table 2, attachment anxiety was positively associated with ED symptoms, even after controlling for EA symptoms. At the same time, attachment avoidance was positively associated with EA symptoms, even after controlling for ED symptoms. The association between attachment anxiety and EA symptoms, was almost completely nullified once ED symptoms were controlled for. Controlling for age and gender did not change these results.

**Table 2:** Standardized maximum likelihood estimates for regressions predicting ED and EA from attachment orientations

	Eating disorder symptoms				Exercise addiction symptoms			
	β	SE	t	p	β	SE	t	p
Attachment anxiety	.22	.05	4.76	< .001	.01	.05	0.24	.811
Attachment avoidance	.004	.05	0.01	.925	.19	.05	4.11	< .001
Eating disorder symptoms					.34	.04	7.59	< .001
Exercise addiction symptoms	.34	.04	7.58	< .001				

Both models are saturated (DF = 0)

In sum, the results support our hypotheses, indicating that attachment anxiety is uniquely related to ED symptoms but not to EA symptoms, whereas attachment avoidance is uniquely related to EA symptoms but not to ED symptoms.

## Discussion

In this study, we investigated the relationship between two closely related psychological pathologies, ED and EA, and their independent associations with IA via distinct IA styles. We surveyed 405 individuals meeting the criteria for recreational exercisers and found that an anxious attachment style was linked to ED but not to EA (supporting H1), whereas an avoidant attachment style showed an association with EA (supporting H2). These results have two main implications: firstly, they provide further evidence for the existence of EA as a distinct pathology that is related to but independent of ED [37, 73]. Secondly, they suggest that IA dimensions may have different trajectories that can potentially lead to the development of different pathological and addictive disorders [41].

Regarding the first implication, differentiating between ED and EA has both practical and theoretical inferences. From a practical perspective, the comorbidity of these two psychological pathologies involves the risk that only one problem be treated [37]. In most cases, ED is treated as it is the better-known of the two disorders and is easier to recognize and identify, potentially neglecting EA and its severe consequences [73]. Theoretical implications suggest that ED and EA may have different underlying mechanisms despite their frequent co-occurrence. While both are associated with attachment styles and emotion regulation deficits, EA has been linked to difficulties in regulating positive emotions, while EDs are more strongly related to difficulties in regulating negative emotions [51]. The emphasis on physical appearance in EDs may also be related to issues pertaining to self-esteem and social approval, while the motivation for exercise in EA may be more related to achieving a sense of control and mastery [39]. These differences highlight the need for different treatment approaches and the importance of distinguishing between the two psychological pathologies.

The second implication is that IA may serve as a significant dispositional risk factor for the development of psychological pathologies, including ED and EA [31]. Given the cross-sectional nature of our study, causality cannot be inferred, however, it suggests a potential association between IA and these pathologies. Furthermore, attachment should not only be dichotomized as secure or insecure but should also be evaluated based on the various dimensions of IA. Despite anxiety and avoidance styles having distinct regulation strategies and exhibiting diverse patterns in relation to disorders, they are frequently treated as interchangeable. Our research findings indicate that an individual's unique rankings on attachment dimensions may give rise to various psychological pathologies and addictions. This highlights the need for further research to explore the specific links between IA styles and different disorders [30, 42, 57, 60, 80].

Regarding individuals with an anxious attachment style, our results suggest that they may be more likely to engage in excessive exercise as a means of addressing body-image concerns. On the other hand, individuals with an avoidant attachment style may exhibit a higher tendency to develop exercise addiction as a coping mechanism for emotional difficulties. However, it is important to note that these interpretations should be made cautiously, considering the limitations of our study and the need for further research to establish the causal nature of these relationships.

The effectiveness of incorporating self-regulation strategies into treatment plans for individuals with IA has been proposed in recent research [34]. Additionally, cognitive-behavioral therapy (CBT) has shown promise in modifying maladaptive beliefs and expectations related to attachment in relationships and reducing cognitive and emotional reactivity [49]. Given the challenges in altering an individual’s attachment style, educating individuals on emotion-focused coping strategies to better manage negative emotions appears to be a more practical treatment approach [12]. Further research should investigate the efficacy of such treatments specifically for individuals with ED and EA.

### Limitations and future research directions

One major limitation of this study is the restricted sample that was included. The criteria for participant selection focused on individuals with an exercise history longer than one year, potentially excluding those who engage in intense exercise behaviors within a shorter timeframe but still demonstrate symptoms of EA. This exclusion of individuals with a shorter exercise history might have led to an underrepresentation of individuals who could potentially score highly for EA. While this limitation does not compromise the validity of the findings within the selected sample, it is important to acknowledge the potential impact on the generalizability of the results. Future research should consider including a broader range of participants with varying exercise histories to obtain a more comprehensive understanding of the relationship between attachment styles, EA, and EDs.

Additionally, this study relied on self-administered questionnaires, which may have introduced social desirability and recall biases. The evaluation tools used were screening tools with no diagnostic validity [7]. To address these issues, future studies could incorporate objective measures of attachment styles during physical activity, such as measuring cortisol levels in the hypothalamic–pituitary–adrenal (HPA) system [68]. An alternative indirect assessment method is the use of narrative techniques, such as the Adult Attachment Interview (AAI [36], and the Adult Attachment Projective Picture System (AAP,[35]. These narrative interviews emphasize mental representations and could provide insight into unconscious defensive processes, a dimension that is neglected by self-report measures.

Future research should also explore how the type of human interaction in exercise affects EA symptoms [25]. Previous research has found that endurance sports, such as running and triathlon, have the highest prevalence of EA symptoms [25]. Moreover, EA symptoms differ among those who participate in team sports and those who exercise individually [48, 73]. Therefore, it is crucial for future research to consider these factors to develop a more comprehensive understanding of the relationship between exercise and attachment styles.

Furthermore, in the context of ED, future research should explore the bidirectional relationship between IA and ED. While previous studies have demonstrated a relationship between IA and ED, the nature of this relationship is not yet fully understood [8, 24]. Therefore, it would be valuable for future research to investigate the directionality and underlying mechanisms of the relationship between IA and ED.

## Conclusion

This study contributes to the growing literature on the associations between attachment styles, EDs, and EA. Our findings indicate a positive association between attachment anxiety and ED symptoms, while the association with EA symptoms was not found to be significant. Conversely, attachment avoidance showed a positive association with EA symptoms but not with ED symptoms. These results suggest a potential link between individuals with an anxious attachment style and a higher likelihood of engaging in excessive exercise to manage body-image concerns. Similarly, individuals with an avoidant attachment style may be more prone to developing EA as a coping mechanism for emotional difficulties. These findings have practical implications for recognizing dispositional risk factors for EA and ED. However, it is important to exercise caution when generalizing the results due to the study’s limitations, and further research should address these limitations to enhance our understanding of the relationship between attachment styles, EA, and EDs.

## Data Availability

Data and materials are available upon request and with permission of Dr. Dalit Lev Arey at dalitlev@mta.ac.il.

## Abbreviations

DSM: Diagnostic and statistical manual of mental disorders
WHO: World Health Organization
ED: Eating disorders
EA: Exercise addiction
IA: Insecure attachment
SR: Self-regulation
CBT: Cognitive behavioral therapy
EAI: Exercise addiction inventory
EAT26: Eating Attitudes Test
ECR: Experiences in Close Relationship Scale
PA: Physical activity
